# Deep prosthetic joint infection: a qualitative study of the impact on patients and their experiences of revision surgery

**DOI:** 10.1136/bmjopen-2015-009495

**Published:** 2015-12-07

**Authors:** Andrew J Moore, Ashley W Blom, Michael R Whitehouse, Rachael Gooberman-Hill

**Affiliations:** Musculoskeletal Research Unit, School of Clinical Sciences, University of Bristol, Southmead Hospital, Bristol, UK

**Keywords:** Joint Prosthesis, Infection, Surgical Revision, Patients, QUALITATIVE RESEARCH

## Abstract

**Objectives:**

Around 1% of patients who have a hip replacement have deep prosthetic joint infection (PJI) afterwards. PJI is often treated with antibiotics plus a single revision operation (1-stage revision), or antibiotics plus a 2-stage revision process involving more than 1 operation. This study aimed to characterise the impact and experience of PJI and treatment on patients, including comparison of 1-stage with 2-stage revision treatment.

**Design:**

Qualitative semistructured interviews with patients who had undergone surgical revision treatment for PJI. Patients were interviewed between 2 weeks and 12 months postdischarge. Data were audio-recorded, transcribed, anonymised and analysed using a thematic approach, with 20% of transcripts double-coded.

**Setting:**

Patients from 5 National Health Service (NHS) orthopaedic departments treating PJI in England and Wales were interviewed in their homes (n=18) or at hospital (n=1).

**Participants:**

19 patients participated (12 men, 7 women, age range 56–88 years, mean age 73.2 years).

**Results:**

Participants reported receiving between 1 and 15 revision operations after their primary joint replacement. Analysis indicated that participants made sense of their experience through reference to 3 key phases: the period of symptom onset, the treatment period and protracted recovery after treatment. By conceptualising their experience in this way, and through themes that emerged in these periods, they conveyed the ordeal that PJI represented. Finally, in light of the challenges of PJI, they described the need for support in all of these phases. 2-stage revision had greater impact on participants’ mobility, and further burdens associated with additional complications.

**Conclusions:**

Deep PJI impacted on all aspects of patients’ lives. 2-stage revision had greater impact than 1-stage revision on participants’ well-being because the time in between revision procedures meant long periods of immobility and related psychological distress. Participants expressed a need for more psychological and rehabilitative support during treatment and long-term recovery.

**Strengths and limitations of this study**

- This study contributes new information about the impact on patients of deep prosthetic joint infection (PJI) and its treatment.
- A sample size of 19 patients allowed for a wide variety of experiences, theoretical saturation and a robust analysis.
- The sample was derived from five National Health Service (NHS) orthopaedic departments ensuring results are transferable to other contexts, although inclusion of further study sites might have raised new issues.
- The use of a cross-sectional study design means there may be patient recall bias as the period between interview and last operation varied from 2 weeks to 12 months though the impact of PJI remains long term.

## Introduction

In the UK in 2013, approximately 80 000 primary hip replacements were performed, the majority of which were for osteoarthritis (91%).^1^ Hip replacements aim to alleviate pain and improve mobility. However, in the UK, around 0.5–1% of patients receiving total hip replacement subsequently develop deep prosthetic joint infection (PJI), a potentially serious and devastating complication.^2^ ^3^

Deep PJI occurs within the joint area rather than at the superficial wound. Infections occurring up to 2 years after surgery are usually acquired during the operation, while infections occurring beyond 2 years are predominantly acquired through or carried in the blood.^4^ Patients with PJI may have diverse symptoms, including inflammation, pain, loss of function, discharge from the surgical wound, fever, nausea and malaise.^4–6^ Left untreated, infections can result in severe pain, joint dislocation, disability and death.

Surgical site infection (SSI) after any type of surgery has been characterised as “an event that inflicted deep suffering and changed the physical, emotional, social and economic aspects of life in extremely negative ways.”^7^ Comparing patient outcomes in those with uncomplicated total joint replacement and those with infected joints, Cahill *et al*^8^ found that outcomes for pain, stiffness, function and mental health and social functioning in those with infection were unfavourable.

Current treatment options for infection after hip replacement may involve non-surgical treatment with antibiotics, with the addition of surgical removal of dead, damaged and infected tissue (debridement). If this is unsuccessful at removing infection then revision surgery is recommended, in which the original prosthesis is removed and replaced. In more established cases of infection, revision surgery may also be the first option for treatment. There are two kinds of revision surgery used to treat an infection, once more conservative treatments have failed. This may be either a one-stage or a two-stage process. In a one-stage revision process, the infected joint and tissue is removed, a new artificial joint is fitted and the patient is treated with antibiotics. A two-stage revision involves two operations. In the first, the infected joint and tissue is removed and a course of antibiotics is given to treat the infection. A temporary implant or ‘spacer’ may be fitted and the patient is without a permanent replacement artificial joint for some months. During this interim period, people have varying degrees of mobility even without a joint. When the infection has been cleared, a new artificial joint is fitted in a second operation usually followed by a further course of antibiotics (box 1).^4^ ^9^ ^10^

Box 1 **One-stage and two-stage revision treatment**

One-stage revision

▸ In a one-stage revision, the infected prosthesis is removed, and the wound debrided (removing infected tissue) before a new replacement prosthesis is fitted, during the same procedure (ie, under the same anaesthetic)

Two-stage revision

▸ In a two-stage revision, the replacement of the prosthesis is delayed, typically for 3–6 months while treating with antibiotics. During this period a ‘spacer’ made of antibiotic loaded cement or a temporary prosthesis is implanted enabling local delivery of antibiotics and to maintain tissue length. Alternatively no spacer is used. The prosthesis is then replaced during a second operation.

Decision-making about which type of revision surgery is complex and involves multiple factors including age, comorbidities, the type of bacteria causing the infection, and the surgeon's expertise and experience with types of revision surgery.

Although previous studies indicate that SSI causes suffering, and that PJI after hip replacement negatively affects outcomes, no research has characterised the impact of PJI and treatment on patients who have undergone hip replacement. This study aims to describe patients’ experiences and the impact of revision treatment for PJI after hip replacement and to compare patient experiences of one-stage with two-stage revision surgery. This information is crucial to the future design of interventions that may improve patients’ well-being and outcomes.

## Methods

### Study design

To explore experiences of PJI, we conducted semistructured interviews with 19 patients who had received either a one-stage or two-stage surgical revision for PJI in the past 12 months.

*Inclusion criteria*: Age 18+ years, experienced PJI and assigned to either one-stage or two-stage revision surgery at one of five participating National Health Service (NHS) orthopaedic departments in the preceding 12 months.

*Exclusion criteria*: Unable to provide informed consent.

### Sampling, recruitment and consent

To reduce recall bias, we only approached patients who had received revision treatment up to 12 months previously. Lists of patients attending outpatient clinics were reviewed by a member of the clinical care team. This team member then examined referral and follow-up letters to identify patients who had received one-stage or two-stage revision treatment for infection after hip arthroplasty in the previous 12 months. Potential participants were sent information packs, and asked to complete and return a reply form to the research team if they were interested in taking part. The researcher (AJM) then contacted potential participants and arranged to visit them to discuss the study and to conduct an interview if they agreed to take part. Immediately before interview, potential participants had the opportunity to ask questions about the study before providing their written consent to participate, including to audio-recording and publication of anonymised quotations. All interviews took place in patients’ homes, except one that took place on hospital premises.

Thirty-four patients were approached and 19 agreed to take part, with the final sample size determined by achievement of saturation during the iterative analysis process, described below. Patients were sampled purposively using phenomenal variation to ensure a roughly equal number of patients who had received either one-stage or two-stage revision surgery.^11^ The sample comprised 9 patients who had undergone one-stage surgical revision and 10 who had undergone two-stage surgical revision, 12 men and 7 women, aged 56–88 years (mean age 73.2 years; table 1).

**Table 1:** Sample characteristics

Pseudonym	Gender	Age	Revision procedure
Frances	Female	68	One stage
Agatha	Female	81	One stage
Simon	Male	83	One stage
Anthony	Male	83	One stage
Rory	Male	78	One stage
Bill	Male	88	One stage
Roger	Male	68	One stage
Jim	Male	56	One stage
Harry	Male	84	One stage
Catherine	Female	67	Two stage
Lorna	Female	69	Two stage
Wendy	Female	60	Two stage
Amelia	Female	84	Two stage
Maggie	Female	69	Two stage
Robert	Male	70	Two stage
David	Male	80	Two stage
Don	Male	69	Two stage
Charles	Male	59	Two stage
Ray	Male	76	Two stage

### Interview process

Interviews took place immediately after consent had been provided. All interviews were conducted by AJM, an experienced qualitative methodologist. Interview topic guides were developed in collaboration with the research unit's patient and public involvement forum.^12^ The interviewer used the topic guide flexibly to ensure that topics were covered but participants also had the chance to discuss issues of particular importance to them. Questions addressed included experience of PJI, experience of revision surgery and care, impact of infection and treatment, and thoughts about recovery and the future. Questions were similar for both treatment groups with the exception of an additional question about the time between operations for those who received two-stage surgery.

Although 9 patients had received one-stage revision treatment, and 10 had received two-stage revision treatment, the recurrent nature of infection meant that 2 patients (Anthony and Francis) had experienced both types of revision treatment. For those patients, their experience of one-stage and two-stage treatment was explored in interviews. Immediately after the interview, field notes were written to record immediate impressions and thoughts about the interview.

Patients were interviewed once. Interviews lasted from 30 to 119 min (mean 64 min), were audio-recorded, transcribed, anonymised and imported into the QSR NVivo qualitative data management software package.^13^

### Analysis

Data were analysed using thematic analysis.^14^ Analysis started with reading and re-reading of transcripts, followed by inductive coding and grouping of coded data into themes and subthemes, with further refinement to ensure internal coherence (fit within the pattern of the theme) and externally for representativeness (fit within the whole data set).^14^ Coding of all transcripts was conducted by the interviewer, with four transcripts independently double-coded by another experienced qualitative researcher (RG-H), and codes discussed, agreed and then applied to the data set with ongoing refinement as needed. Data from participants in the one-stage and two-stage groups were first analysed separately to allow any differences in experiences between individuals and between the groups to become apparent. During analysis, field notes provided context to the interview data, ensuring full understanding of transcribed material. Analysis and data collection took place concurrently, and analysis stopped once saturation had been achieved.^15^ All names are pseudonyms.

## Results

The results showed a wide variation in the experiences of the participants who reported receiving between 1 and 15 revision operations after their primary joint replacement (table 2).

**Table 2:** Treatment trajectory of patients with prosthetic joint infection

Pseudonym and revision type	Treatment trajectory
David 2	Primary hip replacement 2011 Infection symptoms appeared in 2013 Managed for 3–4 months with dressings Two hospital stays to receive intravenous antibiotics, totalling 2 weeks—unsuccessful Referred to Treatment Centre 4—received first stage of a two-stage revision in 2013 Twelve months later still satisfied with ‘temporary’ hip Number of operations after primary hip replacement=1
Catherine 2	Primary hip replacement 2004 Replaced again in 2009 After 10 ‘dislocations’ referred to Treatment Centre 5. Hip replaced again in 2013 After 1 week, Catherine was in severe pain after her replacement and returned to Treatment Centre 5, with infection Two unsuccessful debridement operations Two-stage revision with spacer implant for 10 weeks. Final second stage was September 2014 Number of operations after primary hip replacement=6
Lorna 2	Primary hip replacement 2007—metal on metal Experienced some pain for the following 4 years but thought it was nothing serious. Eventually her leg ‘gave way’ as the top of her femur broke off. Referred to Treatment Centre 5—broken bone fragment removed and new prosthesis inserted Leg began to shorten as stem subsided into medullary cavity as bone was dying Hip replaced again with longer stem at which point an infection was introduced Hip debrided and implant retained—unsuccessful Two-stage revision—during the insertion of the spacer her femur was fractured (only discovered afterwards via X-ray) and subsequently the second stage was done after only 1 week during which screws were inserted to hold femur together Number of operations after primary hip replacement=5
Wendy 2	Fractured hip in 2010 but did not realise until 12 months later when she slipped in the shower and also fractured her femur for which she was X-rayed Emergency hip replacement—infection introduced Low-grade infection went undiagnosed before sepsis occurred. Near fatal Two-stage revision with spacer Spacer dislocated after 2 weeks and was replaced Number of operations after primary hip replacement=3
Robert 2	Primary hip replacement 2004 January 2013 hip subsided as stem sank into medullary cavity. Surgeon revised right hip Surgeon not satisfied so revised again 3 weeks later at which point an infection was introduced. A further washout was unsuccessful. Length of stay in hospital—9 weeks Hickman line inserted so patient could receive antibiotic shots at his local hospital every day for 2 weeks. Antibiotics unsuccessful Referred to Treatment Centre 6 November 2013 first stage of two-stage revision. All but 4 inches of femur removed. Antibiotic beads inserted and retained for 4 months March 2014 second stage Number of operations after primary hip replacement=5
Don 2	Primary hip replacement, April 2009—metal on metal August 2011—revised and debrided due to wear Had three dislocations between October 2011 and January 2012 February 2012 revision again to remedy dislocations (infection introduced) March 2012 debrided—unsuccessful January 2013—first stage of two-stage revision July 2013 further debridement needed April 2014—second stage Interim period of 14 months without spacer Number of operations after primary hip replacement=6
Maggie 2	Primary hip replacement 2005—metal on metal July 2013 revised (infection introduced) January 2014—first stage revision April 2014—second stage revision Fourteen weeks without hip or spacer Number of operations after primary hip replacement=3
Charles 2	Primary hip replacement 2003 December 2012 developed unknown infection. Washout—unsuccessful January 2013—first stage revision with spacer inserted February 2013—complications pulmonary oedema, thoracoscopic drainage October 2013—second stage of revision. Two weeks later hip then dislocated—further revision for dislocation resulted in nerve damage Ten months with spacer Hospitalised for 2 months Number of operations after primary hip replacement=4
Ray 2	Primary hip replacement February 2013—developed infection First stage revision—reoperated after discovering they had fractured his femur when fitting spacer Second stage Number of operations after primary hip replacement=2
Amelia 2	Primary hip replacement 2008 Eight to nine months later revised for dislocation. Extremely painful for a few months as infection had set in after screw broke and hip loosened. January 2013—first stage, no spacer August 2013—second stage Seven months without spacer Number of operations after primary hip replacement=3
Jim 1	Primary hip replacement October 2008 (hip resurfacing) 2013—onset of symptoms of infection. Single stage revision a few days later followed by 9 months of antibiotic treatment Number of operations after primary hip replacement=1
Roger 1	Primary hip replacement 2011 Developed sepsis only weeks later—near fatal Three washout operations—unsuccessful Stayed on antibiotics for 12 months under same surgeon then sent to Treatment Centre 1—turned down the offer of a revision operation for a further 12 months remaining on antibiotics until he could get cover to take care of his father Two years on antibiotics Single stage revision Number of operations after primary hip replacement=4
Harry 1	Left and right primary hip replacements 1992 May 2013 left hip replaced after fall (infection introduced) Single stage revision, unsuccessful Still has infection Antibiotics for 12 months at time of interview Number of operations after primary hip replacement=2
Bill 1	Primary hip replacement 1994 2004 replaced primary (infection introduced) Infection then remains for a further 10 years Unclear how many operations but at least one further revision operation in that time 2013 single stage revision Number of operations after primary hip replacement=approximately 4
Harriet 1	Primary hip replacement April 2011 February 2014—onset of symptoms of infection followed by single stage revision Number of operations after primary hip replacement=1
Rory 1	Primary hip replacement 2001 2005–2006 developed infection, and hip replaced in single stage revision 2012 further revision after femur snapped 2013 onset of symptoms of infection January 2014—single stage revision Number of operations after primary hip replacement=3
Anthony 1	Primary hip replacement right hip 2004 Primary hip replacement left hip 2007 2010 right hip became infected. Revised two-stage revision. Without hip for 3 months. Spacer broke after 2 weeks December 2010—second stage revision of right hip. Had heart failure during hospital admission and subsequent depression. Also diagnosed with leukaemia 2014 infection spread to the left hip. Revised with single stage revision Number of operations after primary hip replacement=3
Simon 1	Primary hip replacement August 2013 September 2013—single stage revision Number of operations after primary hip replacement=1
Francis 1	Primary hip replacement right hip December 2005 Primary hip replacement left hip February 2010 February 2011—diagnosed lump on right hip as bursar Twelve operations (draining and packing) over a period of 18 months October 2012—first stage operation of two-stage revision of the right hip with spacer beads inserted for 3 months January 2013—second stage operation March 2014—infection spread to left hip October 2014—single stage revision of left hip Number of operations after primary hip replacement=15 in total (14 on right hip)

They explained that these included revision for prosthetic joint failure (wear) and complications associated with previous joint surgery (dislocation of prosthesis, femoral fracture associated with surgery) as well as revision for infection. Infection and treatment occurred over periods ranging from 12 months to over 10 years (table 2). Two patients reported that they expected to live indefinitely with infection with lifelong antibiotics despite receiving multiple surgical treatments.

**Figure 1:**
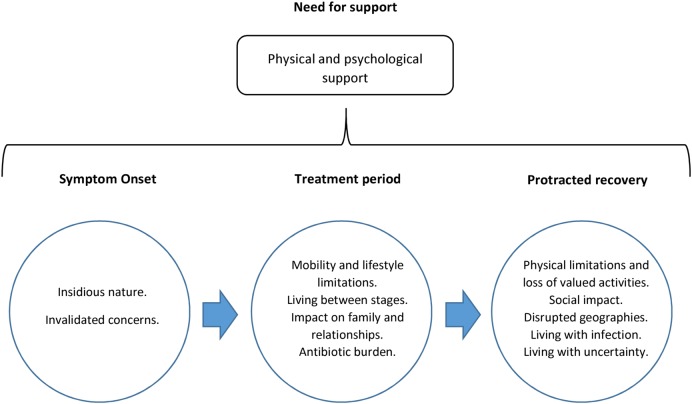
Impact of prosthetic joint infection and revision treatment.

Analysis indicated that participants made sense of their experience through reference to three key phases: the period of symptom onset, the treatment period and protracted recovery after treatment. By conceptualising their experience in this way, and through subthemes that emerged in these phases, they conveyed the ordeal that PJI represented. Finally, in light of the challenges of infection, they described the need for support in all of these phases (figure 1).

All subthemes within the three key phases and those relating to the need for support are described in turn alongside comparisons between the experiences of those receiving one-stage or two-stage revision. Box 2 presents illustrative quotations that correspond with each subtheme.

Box 2 **Illustrative quotes**

**Symptom onset**

1.1 Insidious nature

You wouldn't have thought you would have got an infection after five years […] Out of the blue, that's what I can't understand. (Jim—one stage)

Nobody knew where the infection had come from…I hadn't had any broken skin. I hadn't had an accident of any form, I had no viral problems of any sort. (Rory—one stage)

I'd go out anywhere and everything would be soaking. And I went to [retail store] once and I feel ever so guilty, but I was standing by the counter, and I felt this [laughs] thing go down my leg, and a part of the dressing had just flopped on the floor. (Lorna—two stage)

So they tried the big pump, but that was embarrassing because if my son took us to a café […] And I've sat there and I’ve had to put the tube down me trouser leg because it was partly showing and you could see the blood and all that, all the nasty stuff coming out of the tube into the little box. (Ray—two stage)

You start to feel like, it was a flu-like sickness, and I was trying to fight a virus or something, something that was going to pass, and I was getting worse, and it got to the point where I wasn't even rational and, in fact, two GPs dragged me off and shoved me in an ambulance […] In fact, [surgeon] told me since, he didn’t tell me at the time, “We thought we'd lost you.” (Roger—one stage)

[Nurse] said I don't think you realise how ill you were […] she said “you almost died on us”. (Wendy—two stage)

Infection never crossed my mind because I didn't realise that you could get this in the joint. All I knew was that I had pain […] I really was rolling around, writhing around in agony. I was on very heavy doses of pain relief, cocodamol, oxycodone, I was on morphine. That went on, on and off, for weeks. (Maggie—two stage)

The problem is that exactly one year later, my leg is swollen up and I can barely move. (Rory—one stage)

1.2 Invalidated concerns

I knew there was something seriously wrong. I was going to my GP probably once a fortnight saying, “I can't stand this anymore. I don't seem to be getting any better” […] I knew that the pain was not a muscle pain. I'd been through the procedure once before. It was a most distressing time because nobody seemed to be actually hearing what I was saying.” (Maggie—two stage)

**Treatment period**

2.1 Mobility and lifestyle limitations

I wouldn't say that it's entirely attributable to the infection, but the operation obviously puts severe restrictions, on somebody like me who doesn't want to go on being lame and, it puts severe restrictions on going up mountains and I gave up tennis, I gave up this, I gave up that […] And golf. You know, so those restrictions were just as much connected with the operations and the weaknesses thereof as, as the infection. (Anthony—one and two stage)

I'm in a different situation because I've had seven operations so it's much weaker than most so for a guy who's had his first operation, no infection, just one hip replaced with another; his situation is totally different from mine. He's still got the muscle strength there to keep the hip in place. Mine's all gone. It was eaten away by this pseudo tumour. (Don—two stage)

It's taken a lot of mobility away so that actually takes away entertainment, interests […] It's just the pure reduction in mobility that is the nuisance. It's taken away the bowls, it's taken away the walking. (Roger—one stage)

Oh yeah, we were big walkers, so we were big walkers, then I used to go swimming three times a week, and then I, I, we do our own gardening but obviously I can’t do it now […] I can actually walk now with a stick, but only for short distances and only if I know where I'm going. (Francis—one and two stage)

They put that in and they stitched me all put and everything, and then I had it X-rayed, then they realised that they'd fractured my femur. So they had to open it up again, wire it up. (Ray—two stage)

Fourteen months without a hip joint so it meant that I couldn't drive a car, I couldn't do anything that I'd been used to doing, playing golf or doing anything […] I couldn't do the day-to-day things and walking around the house was difficult because I couldn't carry anything because I was on crutches all the time. (Don—two stage)

As I explained I was looking after my father when this all began—on my own, which made it very difficult during recovery to get that covered and the rest of it […] [surgeon] was trying to drag me into the two-stage and I was fighting for the one-stage. And he looked up the figures, he said success rate drops about 5%. I said, “Well, that's not a bad gamble.” (Roger—one stage)

2.2 Living between stages

Yeah, well you're suddenly reduced, it's quite sudden, suddenly reduced to immobility because they descend on you and take out at the second stage everything, and you're weakened then you have massive antibiotics with drips and things going in intravenous and, er, it, it's, it's, er, it makes you feel very very…down […] Oh, I was desperate you know in terms of…it, it was an awful stage to go through. (Anthony—one and two stage)

Your life is destroyed, absolutely destroyed. There is nothing you can do. You lose your privacy. You lose your dignity. You lose your independence. You have no life. For someone like me who lived a very physically—and I'm a very gregarious person, I would have happily—in fact I would have happily ended it all. I stood at the top of the stairs many times and thought, “If I just went, could I guarantee that this would get me out of this?” because it was that desperate, and I'm a very strong person. (Maggie—two stage)

2.3 Impact on family and relationships (during the treatment period)

The family were visiting me in [Treatment Centre 3], but there is an 80 odd mile round trip. (Bill—one stage)

Your wife becomes a carer, you know? Not least of all having to go 20 odd miles to the hospital every day. And she did, every day. (Rory—one stage) Continued

We did try to go away, but I was so ill we had to come home because I was in such pain. I thought we weren't going to see 51 years because you get to the stage when you hate each other […] you can't get into bed on your own. You can't get out of bed on your own. I had to use a commode for part of the time. I didn't want that. I did not want my husband to see that, or have to do that for me and neither did he. We both hated it, but we had to do it. (Maggie—two stage)

2.4 Antibiotic burden

Oh, they were terrible. It was a hard time to keep food down and things, you know. It’s awful. (Catherine two stage)

Then I started with really bad intense diarrhoea…and of course it's horrible when you're in a main ward; if they don't get to you straight away you've had it. (Francis—one and two stage)

[I] was on really heavy dosage of antibiotics for another eight days […] My bowels, urine and all of that had gone crazy […] My wife and I are married 50 plus years, and I have to have my own room because I'm getting up in the night. (Rory—one stage)

They gave me two choices then […] we can either put you back in the wheelchair for life, or you can take massive gradually decreasing, hopefully, doses of antibiotics, suitable antibiotics and you'll have to keep on taking that, you won’t get any better. So I chose the antibiotics. Which, were remarkably good in that they stabilised me and I've had a reasonable existence ever since. (Anthony—one and two stage)

**Recovery after treatment**

3.1 A changed life—physical limitations and a loss of valued activities

Oh, I find as well that, if I want to go and do anything in the garden, I can only go out and do something for about five minutes, and then my legs are gone […] So, yes, it has changed my life totally, but I'm, I'm not prepared for it to finish my life…I've adapted what I can adapt, and I've still got a life. (Lorna—two stage)

You stop doing things. I was captain of a local snooker club in [place]. I played golf at [place]; all of that's gone. Gave up my golf clubs to the boys next door. Can't play snooker because I'm afraid of pivoting on this leg. It's not worth the risk. (Rory—one stage)

I need to keep moving or go and lie in bed. If I've done anything, I mean I try and keep as fit as I can, but I don't go out walking to keep fit because that's just sheer torture. I do things like try and cut the grass, it might take me about three hours, but I've done something and it's getting you a bit fit. Usually by the time I'm done I'm wiped out, and I usually go to bed. After doing a couple of hours of something I've got to go to bed. (Jim—one stage)

3.2 Social impact—social roles, social life and relationships

It's taken a lot of mobility away so that actually takes away entertainment, interests. (Roger—one stage)

Well we used to go out almost every week […] with our friends, we don’t seem to do that much now, my husband tends to have to do a lot for me now, I mean silly things like you know my feet, my toenails, because when I bend this is hurting me all and everything like that so…(Wendy—two stage)

It's like the tap upstairs. We needed, it was leaking, so we wanted a new tap fitting. I had to get the brother-in-law to put it, where I would have been able to do it myself, but I just cannot bend down and get in to them positions to work now, to do something as simple as change a tap […] And, I mean, I was an engineer […], you know, so it's, but I, I cannot do the simplest of things now. (Robert—two stage)

I spend quite a lot of time in bed now because that's the only way I can cope with everything really. Sometimes it's just better to go to sleep and the day's gone then […] I've got hardly any concentration powers any more. I do snap quite quickly, which I used to be very placid. You've basically got a brain but it's trapped in a useless body. (Jim—one stage)

3.3 Disrupted geographies—moving home, into care

This is a good part of why I'm moving. Not only to get away from the big garden […] you begin to think of taking care of your family and so on. We're moving to an apartment that my wife will be happy in. Even if I'm not there. (Rory—one stage)

We said, you know, ‘what's the chance of getting a stair lift?’ And they said, ‘Off the council, very poor, but you can hire them.’ So I hired it, and that made the difference. That's the difference between having this [the sitting room] as a bedroom and me getting upstairs to use the facilities upstairs. (Robert—two stage)

3.4 Living with infection

It isn't physically limiting; it's just that the awful feeling that it's there and it could well be terminal or degenerate into a terminal condition. Because it's more insidious, the infection, and I think that affected me a lot […] you feel definitely much degraded. (Anthony—one and two stage)

I kind of worked like hell at them [hip replacements] when I'm recuperating and do all the exercises […] So I'm working at it all the time […] I can’t do that with the infection. You're powerless; that's the difference to me. (Anthony—one and two stage)

3.5 Living with Uncertainty and concerns about the future

Well for example in the back of my head I'm thinking September, October for me this year it's gonna be another checkpoint. Because two successive years these problems have arisen in that period; roughly six to eight months after the operation. If I got through this early autumn, I can say this whole thing is a success. (Rory—one stage)

So, yeah, I do worry about it getting re-infected. And I still don't feel completely I'm out of the woods yet, and yet it's almost a year now, you know. I'd like—you, sort of, think, ‘When can you relax? (Lorna—two stage)

But that's the only thing I really panic about now, is getting another infection, because I don't know where he'd go from there. It'd be leg off time then, wouldn't it? [laughs] (Lorna—two stage)

I should hate to get to the situation where I've got to have a leg off. I'd rather die. So the most important thing now is to try and keep two legs. (Harry—one stage)

And then [Consultant H] is whispering to me, “Please don't fall over; stay on a stick. If you fall we've got nothing left to repair.” (Rory—one stage)

And I know both of my knees are on the way out…And I'm frightened that all this is gonna…make one of the knees go, which, obviously, would mean another trip into hospital, and another bloody operation, which is the last thing I want. (Robert—two stage) Continued

That's my biggest fear because it's a painful experience I can tell you because I did it three times and it's the most excruciating painful experience you can have […] I'm still living in fear of doing things right, after the operation you know? (Don—two stage)

With this pain, he said it is going to go away. If it goes away, that's fine. I wouldn't want to see myself perpetually having to take painkillers all the time, all the time. (Charles—two stage)

Well just to keep going and not to end up in a wheelchair, because then I am so dependent on somebody coming along to push me here, there and everywhere. So if I can keep mobile and keep well away from the wheelchair, that is my greatest concern. (Amelia—two stage)

Well I just hope that I can start to walk properly and you know get back to a normal social life and things like that…And able to do things in the house you know like simple things like I used to change a light bulb. (Wendy—two stage)

I'm [age], training with people…Well I expect 10 year olds could run rings around me on a computer, and that's the sort of skill I've got and I've got to compete against people like that to try and get a job. And it’s just battering you, one thing after another. That's the pressure, no money and can't get a job. If I knew I could get back to building then I'd be happy, but I've got to be realistic. (Jim—one stage)

The need for support

A bit more preparation for, what could go wrong. I mean, they did warn me, as I said, that, it is only a weak stuff [the spacer] but you don't take that in fully. I think a little more counselling initially, a little more, preparation. (Anthony—one and two stage)

Yeah, well you're suddenly reduced, it's quite sudden, suddenly reduced to immobility because they descend on you and take out at the second stage everything, and you're weakened then you have massive antibiotics with drips and things going in intravenous and it makes you feel very very…down […] Oh, I was desperate you know in terms of…it, it was an awful stage to go through […] I think that's the one period that…some form of treatment or advice or counselling or something needs to be really improved. I don't know what other people have gone through with this, whether they have the cracking and the crumbling that I had, because that was the awful part and I think, you know, I was reduced to almost complete immobility. (Anthony—one and two stage)

I mean this is the one thing that I highlighted to [surgeon] […] what you need is to be able to pick up the phone and say to someone, “I don't know how I'm going to get through the rest of the day.” You know, “I don't see any end to this. I don't know how it's ever going to get better,” because you can't. Everyday, 24 hours is a long, long time, you know, you lie awake in the night. It's a long and lonely existence. And I think if you could just—you cannot offload onto your family, because…[becoming tearful] they're caring for you. It's very distressing for all of them. Your family, you can't just go to them and say, “I want to end this. Get me out of it. I can't stand it anymore” […] what I would have liked most, is some person that didn't know me, that I could just ring up and offload and say, “I'm really fed up to the back teeth with this.” (Maggie—two stage)

I had decided that from the *first* morning that I didn’t need her but I didn’t stop her. I let her come the four days just to have somebody to talk to. (Catherine—two stage)

I don't know how anyone would cope, that didn't have someone to care for them, didn't have a partner or a member of their family who could look after them. I don't know how they would manage, either physically or emotionally. (Maggie—two stage)

I'm not really happy with the physio, actually. Erm, because it's, like, you go and it's, like, you've got six sessions, and they're going, ‘Oh, well, that's coming on good. That's, but you're finished now.’ (Robert—two stage)

When I had my bypass I came out after the operation, and I had problems [becoming tearful]. I had, er, like, medical depression. Erm, and I went to see a, a psychologist. It was arranged. I had to wait […] but I went to see a psychologist, and she asked a lot of questions. Erm, and I had to, sort of, answer the questions and try what she said. Erm, I think if I'd had somebody to talk to and answer some of the things I was a little bit doubtful about myself, it would have helped. (Robert—two stage)

### Symptom onset

#### Insidious nature

Infection occurred either after a primary hip replacement, or after revision surgery for instance for worn, loose or dislocated prosthesis. Infection could occur immediately or shortly after the operation, but some participants developed infection many years later, which they found particularly unsettling. Signs and symptoms included severe pain, red, inflamed and sore wounds, and abscesses which burst and wept pus or fluids, and could be painful but also socially embarrassing. For instance, Ray initially had a pump fitted to drain fluid from his wound, but its visibility made social situations awkward.

Low-grade infections presented as a more general sickness and malaise which could, if not recognised early on, lead to severe sepsis. Both Roger and Wendy described how infection had started as flu-like symptoms, the importance of which was not initially realised either by themselves or health professionals. Three months after her primary operation Wendy lost consciousness at home and had been rushed in to hospital. She had no memory of the five initial days at hospital, but had later been told by a nurse that she had nearly died due to the infection. Other patients reported more overt symptoms at onset such as agonising pain and a loss in mobility, although they did not initially associate these with PJI.

#### Invalidated concerns

Some patients expressed distress that their early concerns that something was wrong were not acknowledged, feeling that they had not been taken seriously. They felt that earlier identification of infection would have lessened the duration for which they had endured painful and debilitating symptoms.

### The treatment period

#### Mobility and lifestyle limitations

Participants described loss of physical function and mobility in relation to both the infection and the surgical treatment that they received. The number of treatments which patients received had a proportionate deleterious effect on the strength and stability of their joint due to the removal of infected bone and tissues. This was particularly the case for those who had multiple previous revisions that could include both one-stage and two-stage procedures. Both procedures affected patients’ physical mobility, posing restrictions on walking ability and day-to-day activities.

However, patients who had undergone two-stage revisions reported additional challenges to their mobility. Once the infected prosthesis was removed, patients had no functioning hip joint. Some patients were fitted with a ‘spacer’ (box 1). Others did not receive a spacer. Four of eight patients who had a spacer fitted experienced complications such as fracture or dislocation of the spacer which caused further pain, discomfort and immobility. In some cases, this necessitated further surgery to replace the spacer. During the fitting of their spacers Lorna and Ray experienced fractured femurs that required further surgery. Others who had no spacer found their level of mobility further reduced. Although some described how they had managed on crutches, older, frailer patients were immobile for long periods, which posed more burden on their carers. One patient who cared for his father requested a one-stage revision to avoid a more prolonged period of immobility, in spite of his surgeon's recommendation of two-stage revision.

#### Living between stages

The major difference in the accounts of those who received a one-stage revision and those who received a two-stage revision was the time between operations—the interim period—during which patients had a spacer device fitted or no spacer for a period of 10 weeks to 14 months. During this period, patients experienced considerable physical and psychological difficulties.

The interim period also increased the burden of care on partner and family, and sometimes meant that the patient stayed in hospital until their hip was replaced or their home environment was adapted. For older and more immobile participants and their families, this period was particularly challenging, for instance, Amelia was without a hip or spacer for 6 months, which her family found particularly difficult to manage.

The sudden reduction in mobility during the interim period also had profound psychological effects. A poignant example is Maggie whose husband cared for her during this time. She described physical suffering, loss of dignity and independence and the realisation that her life had changed suddenly and negatively. This led her to consider suicide during the interim period.

#### Impact on family and relationships (during the treatment period)

The infection and revision treatment also impacted on family and personal relationships to varying degrees. Costs in time and travel to see patients in hospital, and the burden of caring for someone suddenly rendered immobile for extended periods strained personal relationships. This was particularly the case for those who underwent a two-stage revision when their immobility was extended during the period between procedures. Strain resulted from managing logistics (travel to and from hospital) and the emotional challenge of receiving support for personal hygiene tasks, leading to feelings of indignity and sudden dependence. This was strongly felt by those who had been particularly independent before the infection.

#### Antibiotic burden

Participants reported mixed experiences of antibiotics used for treating infection regardless of which type of revision surgery they had received. While some reported no adverse effects, others felt the antibiotics had caused considerable and often distressing side effects such as diarrhoea and sensory disturbances. Some attributed ongoing problems to previous antibiotic use, with problems including stomach ulcers and increased need to urinate, even after they had stopped taking the medication. However, two patients whose infection could not be eradicated described antibiotics as life sustaining, explaining that antibiotics prevented them from losing a limb or dying of sepsis.

### Protracted recovery after treatment

#### A changed life: physical limitations and a loss of valued activities

Although patients adapted to varying degrees, infection and treatment affected their ability to carry out day-to-day activities, with short-term and long-term implications. Maintaining homes and leisure activities became almost impossible. Those living alone often relinquished valued activities and changed how they managed periods of activity and rest because of reduced mobility.

#### Social impact: roles and relationships

Losses of physical ability also impacted on social relationships. Compromised social roles and changes to social identities caused frustration and other issues. For instance, Robert partly attributed his depression to his need to ask others to perform tasks that he felt were simple to a man of his former abilities. Jim had to give up his manually skilled profession after infection, which created financial difficulties. He found it hard to retrain for different work because of reduced concentration and a new propensity for his mood to ‘snap’, which he suggested was linked to frustration about sudden physical disability.

#### Disrupted geographies: moving home or into care

With losses in mobility and physical capacity, some participants moved into care homes or smaller residences. For instance, at the time of interview Rory was in the process of moving from a house to an apartment. He explained that he could no longer manage his garden and he wanted a more manageable home for his wife as she took on more caring responsibilities, and in the event of his death.

Participants also changed how they configured their homes. Many had turned a downstairs room into a bedroom during their treatment period. However, the ongoing effects of infection and treatment on their mobility meant that some could no longer manage their stairs safely and they continued to sleep downstairs in the longer term.

#### Living with infection

Two participants had infection that could not be eradicated and took antibiotics every day. The ongoing presence of infection had psychological consequences and they lived in fear of their infection becoming unmanageable. One participant described his experience of recovery from surgery in terms of powerlessness, contrasting this with his experience of the original operation when he had been able to improve with physiotherapy exercises.

#### Living with uncertainty and concerns about the future

At the time of the interview, patients were in a period of recovery, some up to 12 months after treatment (table 2). Participants who had undergone one-stage and those who had undergone two-stage revision lived with uncertainty about the recurrence of infection. There was little difference between the concerns of participants in both groups. Some participants found it difficult to foresee a time when they could relax.

Those who had large sections of bone removed during previous revision surgery expressed concern that recurrence of infection could lead to amputation. Others described a reluctance to have other painful joints replaced for fear of further infection. Participants also found it hard to focus on the future if they had nerve damage or ongoing pain. Those who had experienced dislocations also lived with a fear that it may happen again, which impacted on how much they could plan to do in the future. Also, concerns about the future and regaining or maintaining some independence were particularly related to issues of mobility, self-care and financial well-being.

### The need for support

Participants suggested that there were a number of areas in which they needed support during each of the phases.

All participants expressed an absence of and need for psychological support, but this was particularly pronounced among those who had received a two-stage revision. Participants described a need for better preparedness for the interim period, particularly with focus on the possible impact on family and partners. Those living alone were particularly vulnerable to loneliness and isolation. Some took steps to reduce this, but used their own ingenuity to do so rather than receiving planned psychological support. For instance Catherine let a carer visit her for the first 4 days after discharge from hospital, just to have ‘someone to talk to’.

Participants would also have liked to have had more knowledge about the physical challenges of infection and revision. This was particularly evident among participants who had received both revisions. For instance, during the interim period, Anthony's spacer had broken, leaving him ‘suddenly reduced to immobility’, feeling ‘weakened’ and ‘desperate’. He explained how he had not appreciated the fragility of the spacer and that pretreatment counselling would have better prepared him for the physical and psychological aspects.

The physical and psychological impact of infection is entangled with each other: physical implications of infection and treatment are linked to distress, concern and uncertainty. For patients recovering from infection, the need for support in all of these areas also persists beyond the treatment period, with participants suggesting a need for more physical support in order to reduce psychological problems such as depression.

## Discussion

The insidious onset of symptoms and difficulty in diagnosing deep PJI meant that patients experienced uncertainty, anxiety and fear. Symptoms could be overt, painful and visible, or less obvious and therefore overlooked by patients or clinicians. Some patients felt that their early concerns about symptoms were not heeded by clinicians. During recovery after treatment, ongoing anxiety was caused by uncertainty about future return of infection that might result in further major surgery and possibly amputation. Others lived in fear of dislocating their less stable hip joint. Patients’ revision histories were often complex, extending over many years and many had already received multiple revisions (sometimes both one-stage and two-stage) prior to their participation in the study, which is testament to the difficulty in eradicating infection. Patients experienced pain associated with treatment and complications, and this could persist long after treatment. Sudden and prolonged immobility affected patients both physically and psychologically.

The major difference between the two revision types was that two-stage revisions imposed more treatment burden on patients and families, often due to complications associated with the interim period. The sudden and immense negative change in patients’ lives, the loss in mobility, sense of disablement and ‘degradation’ had a deep psychological impact with some patients reporting depression and suicidal thoughts. Treatment impacted on family and personal relationships to varying degrees. Some participants coped despite the new burden of care, while others reported a strain on personal relationships when their partner suddenly also became their carer. The impact was so great for some patients that they gave up manually skilled employment, which presented financial concerns. Others moved into more manageable homes or residential care homes.

### Strengths and weaknesses of the study

Although we acknowledge that inclusion of future study sites might have produced additional findings, the sample size of 19 patients, derived from five NHS orthopaedic departments across the UK, and the achievement of saturation provides confidence that the findings are transferable to similar contexts.^15^ In addition, these allowed us to address fully the research aims. In the analysis process, double coding ensured that the analysis was rigorous and based on a robust coding framework. Finally, although use of a cross-sectional study design meant that participants were asked to recall their previous experiences and this might have introduced some recall bias, the inclusion of patients at a range of time points between discharge and the interview (2 weeks to 12 months) meant that the study was able to elicit experiences and support needs relating to onset, treatment and the longer term.

### Comparison with other studies

To our knowledge, this is the first study to explore the impact of one-stage and two-stage revision treatment for deep PJI after hip replacement. Andersson *et al*^7^ explored more general patient experiences of SSIs (abdominal surgery, hip and knee replacements, coronary bypass or hysterectomy). Our study resonates with their findings that infection impacts on physical, social and emotional aspects of everyday life. Andersson also reports that patients with SSI experience feelings of insecurity, pain, and felt their concerns were not taken seriously during the onset of symptoms. Patients’ concerns about reinfection were well founded as it is known that around 10% of surgical revisions for infected hip prosthesis become reinfected within 2 years.^16^ Other studies have shown that general SSIs and PJI lead to considerable reductions in health-related quality of life, and negatively affect both physical and psychological outcomes while increasing healthcare costs by as much as 3.6 times greater than that of a primary total hip replacement.^8^ ^17–19^ The use of and complications associated with spacers were a particular challenge for patients within this study. Complications with spacers appear to be relatively common, despite their beneficial use in maintaining tissue length and function, and on study of 88 spacer implants showed an overall complication rate of 58.5% including 15 dislocations and 9 spacer fractures.^20^ Previous research comparing one-stage and two-stage revision treatment has mainly focused on clinical outcomes and reinfection rates and this is the first study to compare patient experiences of these treatments in terms of impact.^16^ ^21^ While reinfection rates are considered to be similar for both revision treatments, we show that the impact on patients can vary greatly.

### Implications for clinicians and policymakers

On the basis of our findings, we suggest that healthcare professionals (surgeons, general practitioners and nurses) focus on optimising education and supportive care strategies to enable earlier recognition of signs and symptoms of infection. An increased vigilance for recent arthroplasty patients and more consideration of their concerns should be encouraged. Patients expressed a requirement for more supportive interventions both during revision treatment (eg, counselling and peer support), and in the longer term (eg, physical rehabilitation and reassurance/active monitoring) as the impact of PJI can persist long after surgical treatment. We recommend future research focuses on designing and evaluating improved care strategies for people with PJI. We are also conducting further research to explore decision-making and preferences for type of revision treatment.^22^
